# P3H4 Promotes Malignant Progression of Lung Adenocarcinoma via Interaction with EGFR

**DOI:** 10.3390/cancers14133243

**Published:** 2022-07-01

**Authors:** Chen Fang, Yingkuan Liang, Yong Huang, Dong Jiang, Jiaxi Li, Haitao Ma, Lingchuan Guo, Wei Jiang, Yu Feng

**Affiliations:** 1 Department of Thoracic Surgery, The First Affiliated Hospital of Soochow University, Suzhou 215000, China; fangchensuda@163.com (C.F.); yingkuanliang@foxmail.com (Y.L.); jd159@163.com (D.J.); 471698023@qq.com (J.L.); mht7403@163.com (H.M.); 2 Department of Thoracic Surgery, Haimen People’s Hospital, Nantong 226100, China; jimei9688@163.com; 3 Department of Pathology, The First Affiliated Hospital of Soochow University, Suzhou 215000, China; szglc@hotmail.com; 4 Department of Thoracic Surgery, Dushu Lake Hospital Affiliated to Soochow University, Suzhou 215000, China

**Keywords:** lung adenocarcinoma, P3H4, EGFR, metabolism

## Abstract

**Simple Summary:**

Lung adenocarcinoma (LUAD) is the most common histologic subtype of lung cancer. Studies have shown that P3H4 is a key gene underlying the malignant progression of LUAD. A potential biomarker and therapeutic target, P3H4 is involved in various cancers, but its molecular mechanism in LUAD remains unclear. Based on a series of experiments, we found that it significantly promoted the metastasis and proliferation of LUAD in vivo and in vitro.

**Abstract:**

Lung cancer is associated with the greatest number of cancer-related deaths worldwide. Lung adenocarcinoma (LUAD) accounts for 85% of all cases of lung cancer. Despite recent advances in treatment, the 5-year survival rate remains less than 15%. Thus, the diagnostic and therapeutic role of LUAD remain to be further studied. The prolyl 3-hydroxylase family member 4 (P3H4) is involved in various cancers, but little is known about its role in LUAD. Our study demonstrated that the P3H4 gene was upregulated in LUAD. Clinically, the expression of P3H4 was positively correlated with an advanced TNM stage and shorter survival. Functionally, P3H4 plays a significant role in the metastasis and proliferation of LUAD both in vitro and in vivo. Mechanistically, P3H4 might interact with EGFR to regulate the metabolic substances. Our study indicated that P3H4 is a critical gene in the malignant progression of LUAD and represents a potential biomarker and therapeutic target.

## 1. Introduction

Lung cancer (LC) is one of the most frequently diagnosed cancers and the leading cause of cancer-related deaths worldwide [1], with an estimated 2.20 million new cases and 1.79 million deaths reported each year [2]. According to histological classification, LC was mainly divided into small cell lung cancer (SCLC) and non-small cell lung cancer (NSCLC) (about 85%) [3,4,5]. Lung adenocarcinoma (LUAD) is the most common histologic subtype and accounts for more than 40% of lung cancer incidence [3]. Due to the popularization of low-dose spiral computer tomography (CT), the detection rate of lung cancer has been further improved [6]. Substantial progress has been made in the treatment of advanced-stage lung cancer (including immunotherapy and targeted therapy) in recent years; however, the 5-year overall survival rate is still not high [7,8]. Therefore, it is of great importance to understand the signaling pathways and molecular mechanisms of LUAD and identify new biomarkers, which may improve the diagnostic rate of early lung cancer and increase the treatment options available for LUAD.

The prolyl 3-hydroxylase family member 4 (P3H4, alias SC65) is a nucleoprotein originally identified as an autoantigen in interstitial cystitis [9]. Gruenwald et al. reported that P3H4 was also an endoplasmic reticulum protein. P3H4 has been found to regulate bone metabolism by modifying the collagen prolyl-hydroxylation after transcription [10]. Heard et al. reported that P3H4 may form a complex with prolyl 3-hydroxylase 3 (P3H3) and bind with enzymes and cyclophilin B to regulate the activity of lysyl-hydroxylase 1 in collagen synthesis [11]. P3H4 has been identified in various cancers. P3H4 promoted cell proliferation, cell cycle, migration and invasion in the bladder [12] and was associated with poor prognosis in bladder cancer [13]. Comtesse et al. reported that patients with meningioma carry antibodies against P3H4, which may represent a new diagnostic and therapeutic target in meningioma [14]. In lung cancer, P3H4 overexpression was identified as a prognostic risk factor; however, no functional or mechanistic study has been reported to date [15]. Overall, P3H4 is involved in a variety of physiological and pathological processes. However, the role of P3H4 in NSCLC remains unknown.

In this study, we demonstrated that the P3H4 gene was upregulated in LUAD and promoted its migration, invasion and proliferation. Mechanistically, P3H4 might interact with EGFR to regulate the metabolic substances to promote malignant progression. P3H4 is a potential biomarker and therapeutic target.

## 2. Materials and Methods

### 2.1. Gene Expression Datasets

A total of 521 samples with lung adenocarcinoma (LUAD) were downloaded from The Cancer Genome Atlas (TCGA) database, including 58 samples of paired tissues (cancer and para-cancer) of RNAseq data, 515 cancer samples containing RNAseq data and 59 normal tissues. The comparative gene expression of LUAD and normal tissues was obtained from the GEO database (GSE40275).

### 2.2. Cell Lines and Culture Conditions

All cell lines (lung squamous cell carcinoma cell line SK-MES-1, lung adenocarcinoma cell lines A549, NCI-H460, NCI-H1299 and normal human bronchial epithelium cell line BEAS-2B) were purchased from the Chinese Academy of Sciences Cell Bank (Shanghai, China). SK-MES-1 was cultured in DMEM, while A549, NCI-H460, NCI-H1299 and BEAS-2B were cultured in PRIM 1640 supplemented with 10% fetal bovine serum (FBS) (Ausbian, Adelaide, Australia) and 100 IU/mL penicillin/streptomycin (Gibco, Waltham, MA, USA), followed by incubation in a humidified chamber at 37 °C in the presence of 5% CO_2_. In the experiment, cells in the logarithmic growth phase were selected for subsequent analysis.

### 2.3. Reverse Transcription-Quantitative PCR (RT-qPCR)

TRIzol was used to extract RNA, based on the manufacturer’s instructions (Thermo Fisher Scientific, Waltham, MA, USA). cDNA was prepared via reverse transcription using random hexamers (Takara, Dalian, Liaoning, Dalian, China). The amplification of mRNA was carried out using PrimeScript RT Master Mix (Takara, Dalian, Liaoning, Dalian, China) and analysis was performed using StepOnePlus™ Real-Time PCR System (Applied Biosystems, Waltham, MA, USA) and the results were normalized to GAPDH expression. Primer sequences of P3H4 and GAPDH were as follows: P3H4 forward, 5′-TCTACCCGGCCATAGCAGATC-3′ and reverse, 5′-TTGTCCACGAAGTAGCCACCC-3′, product length: 100 bp; GAPDH forward, 5′-TGACTTCAACAGCGACACCCA-3′ and reverse, 5′-CACCCTGTTGCTGTAGCCAAA-3′, product length: 121 bp. The relative expression of each sample gene was analyzed via the 2^−ΔΔCt^ method. In order to ensure the accuracy of quantification, each sample was analyzed in triplicate.

### 2.4. Construction of P3H4 RNA Interference Lentiviral Vector

Multiple 19–21 nt RNA interference target sequences were designed using the P3H4 gene as the template. Following evaluation and determination using the design software, CATGTACCTGCAGTCAGAT was selected as the interference target, and appropriate restriction enzyme digestion sites were added at both ends. The synthesized DNA was cloned into the lentiviral expression vector hU6—MCS-CMV-EGFP to complete the vector construction. The connected product was transformed into *Escherichia coli*-competent cells, and positive clones with correct results of identification were preserved. Plasmids were extracted from the positive clones, according to the EndoFree Maxi Plasmid Kit specifications. The vector plasmid carrying the target gene P3H4, and the virus packaging auxiliary plasmids Helper1.0 and Helper2.0 were co-transfected into 293T cells. The virus was harvested 48 to 72 h after transfection, and finally filtered with a 0.45 μm filter.

### 2.5. Western Blotting Analysis

Western blotting analysis was performed to detect the expression of P3H4 protein (SC65) in transfected A549 and NCI-H1299 cells. Cells were lysed with RIPA buffer (Beyotime Biotechnology, Shanghai, China). BCA Protein Assay Kit (Beyotime Biotechnology) was used to determine the total protein concentration. β-actin was used as an internal reference. A total of 50 μg of target protein was loaded onto 10% SDS-PAGE in different lanes, and then transferred to PVDF membrane. Subsequently, the PVDF membrane was blocked at room temperature for 1 h or overnight at 4 °C with blocking solution (TBST solution of 5% skimmed milk). The PVDF membrane was incubated with primary antibodies including anti-P3H4 (1:500); 15288-1-AP; Proteintech, Rosemont, IL, USA) and anti-β-actin (1:5000; sc-69879; Santa Cruz Biotechnology, Dallas, TX, USA) at room temperature for 2 h or overnight at 4 °C. The membrane was then incubated with a secondary antibody such as goat anti-rabbit IgG (HRP-linked goat anti-rabbit IgG, 1:10,000, # 7074, CST) or goat anti-mouse IgG (HRP-linked goat anti-mouse IgG, 1:10,000, # 7076, CST) for 1.5 h at room temperature. Finally, X-ray analysis was performed using 20× LumiGLO^®^ reagent and 20× Peroxide # 7003 kit (CST). The experiment was repeated three times.

### 2.6. Cell Proliferation

A549 cells and NCI-H1299 cells in the logarithmic growth phase were selected. The fluorescence of these cells was detected using the Celigo Imaging Cytometry System version 2.0 (Nexcelom Bioscience, Lawrence, MA, USA) and the number of cells was automatically calculated. MTT assay kit (Genview, Houston, TX, USA) was used to detect cell proliferation. According to the manufacturer’s instructions, the cells were inoculated into 96-well plates at a density of 1500 cells/well, cultured continuously and analyzed for 5 days, following treatment with MTT for 4 h. Subsequently, the optical density (OD) was measured at 490 nm using a microplate reader (Tecan Infinite, Männedorf, Switzerland). Each experiment was repeated three times.

### 2.7. Cellular Apoptosis Assay

A549 and NCI-H1299 cells transfected with shP3H4 or shCtrl were seeded into 6-well plates at a density of 2 mL/well. The cells were passaged on day 3 after infection and detected at 85% confluency on day 5. The apoptotic cells were determined using Annexin V-APC apoptosis detection kit (eBioscience, Waltham, MA, USA) and flow cytometry (FACS calibur; BD Biosciences, Franklin Lakes, NJ, USA), according to the manufacturer’s instructions. The percentage of apoptotic cells was assessed using ModFit LT version 5.0 software (BD, Topsham, ME, USA).

### 2.8. Colony Formation Assays

A549 and NCI-H1299 cells transfected with sh-P3H4 or shCtrl were seeded in 6-well plates at a density of 1000 cells/well 3 days after transfection. The culture medium was replaced every 3 days for 8 days. The cultured cells were fixed with 4% paraformaldehyde for 30 to 60 min, and stained with 0.1% crystal violet for 10 to 20 min (both at room temperature). Each colony of more than 50 cells was manually counted under an optical microscope, and the magnification was ×40. Each experiment was repeated three times.

### 2.9. Wound Healing Assay

Cell migration was evaluated via wound healing experiment. Lentivirus-transfected A549 and NCI-H1299 cells were seeded in 96-well plates at a density greater than 90% confluency. After 24 h of culture, scratches were formed by a scratch tester, and the old medium was replaced with a medium containing 1% FBS. The scratches were monitored at 0, 24 and 48 h. The migration area was analyzed using Celigo Imaging Cytometry System version 2.0 (Nexcelom Bioscience, Lawrence, MA, USA). Each experiment was repeated three times.

### 2.10. Transwell Assay

Transwell assay was used to detect the migration of cells. The upper chamber was filled with serum-free DMEM medium containing 5 × 10^4^ stable transfected cells. The DMEM medium containing 30% FBS was added to the lower chamber. After 16 h of culture, the cells in the lower chamber were stained with Giemsa (Sigma-Aldrich, St. Louis, MI, USA) and counted under a microscope. The experiment was repeated three times.

### 2.11. Animal Experiments

Twenty 4- to 5-week-old BALB/c nude mice were divided into two groups: A549 P3H4 KD and A549 NC. Subcutaneous tumor-bearing nude mouse models were developed by injecting the transfected A549 cells into the axillae of nude mice at a dose of 7 × 10^6^. The caudal vein metastasis model was constructed by injecting 2 × 10^6^ cells into the caudal vein of nude mice. The body weight and tumor size (length and width) of subcutaneous tumor-bearing nude mice were measured and recorded one month later. The weights of nude mice were measured on the second day after the establishment of the metastatic tumor model, and then every two weeks thereafter, followed by imaging with an in vivo biofluorescence imaging system (PerkinElmer, Beaconsfield, UK). The results were collected and the data processed.

### 2.12. Protein-Protein Interaction (PPI) Network Construction

The over-expression lentivirus (OE-P3H4) fused with 3 × FLAG tag (3 × FLAG-bait) at the C-terminal of P3H4 gene was constructed. The FLAG expression by stable cell lines was detected via Western blot, with β-actin as the internal reference. The 3 × FLAG-target interaction complex was purified via coimmunoprecipitation (co-IP). The overall process was started by culturing a large number of NC and OE stable cell lines, and more than 80% of the confluent cells were collected. RIPA lysis buffer was used to lyse cells and extract total protein, and the protein content was determined by the BCA method. Similar amounts of two groups of proteins were analyzed via Co-IP with FLAG-beads (Sigma-Aldrich, St. Louis, MI, USA). The Co-IP samples were subjected to SDS-PAGE electrophoresis and Coomassie blue staining. The proteins in each sample were digested with trypsin, and the peptides formed by enzymatic hydrolysis were identified by LC-MS (Thermo Fisher Scientific, Waltham, MA, USA). The mass spectrometry data of each sample were analyzed using the PD/MASCOT software to identify each of the proteins. The specific protein of OE group was analyzed via bioinformatics analysis.

### 2.13. Statistical Analysis

SPSS 25.0 software was used to perform all statistical analyses. Chi-square test or Fisher’s exact test were used to assess qualitative variables. Student’s *t*-test was used to analyze data showing normal distribution. Non-parametric test was used to analyze variables with abnormal distribution. Analysis of variance (ANOVA) was used for comparing the differences between groups. Correlation analysis was performed using the Pearson correlation coefficient method. The ROC curve was calculated to determine the sensitivity and specificity. The findings are indicated as the means ± standard deviation (SD). All statistical tests were two-sided, and the *p* value < 0.05 was deemed statistically significant.

## 3. Results

### 3.1. P3H4 Showed Increased Expression in LUAD

**Figure 1:**
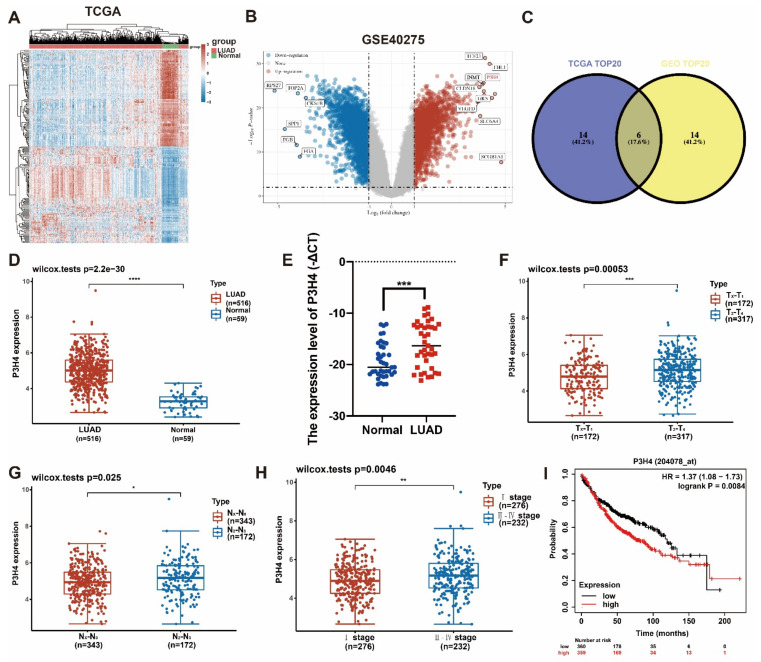
Screening of P3H4 expression in LUAD. (**A**) The heatmap indicates the differential expression of genes between lung adenocarcinoma and normal tissues derived from TCGA database. (**B**) The volcano plot demonstrates the differentially expressed genes among LUAD and normal tissues derived from GSE40275 dataset. (**C**) Venn diagram reveals the six common candidate genes in the two data files. (**D**,**E**) P3H4 was upregulated in LUAD both in TCGA and our cohort. (**F**–**H**) The clinical characteristics of LUAD in samples from TCGA indicate significant upregulation of P3H4 in advanced clinical stages. (**I**) The Kaplan–Meier survival curves show that patients with a high expression P3H4 in LUAD showed shorter overall survival. * *p* < 0.05, ** *p* < 0.01, *** *p* < 0.001, **** *p* < 0.0001.

In order to identify the differential genes with robust biological functions in LUAD, the LUAD gene expression files in both TCGA and GEO (GSE40275) were analyzed (Figure 1A,B). The top 20 differentially expressed genes of two datasets were selected. Six common genes were identified in the two groups (Figure 1C). Based on a comprehensive analysis of the clinical characteristics, P3H4 was identified as the differential gene that was most significantly correlated with malignant progression in LUAD. In both TCGA and our own cohort, P3H4 was significantly upregulated in LUAD compared with normal tissues (Figure 1D,E). In TCGA dataset, P3H4 was positively correlated with advanced LUAD tumor size (T stage) and lymph node metastasis (N stage) (Figure 1F,G). P3H4 was highly expressed in advanced Tumor, Node and Metastasis (TNM) clinical stages of LUAD (Figure 1F). Further, the Kaplan–Meier survival curves indicated that LUAD patients with decreased P3H4 expression showed better survival (Figure 1I).

Thus, P3H4 is a key gene involved in the malignant progression of LUAD.

### 3.2. The Malignant Progression of LUAD Was Suppressed by P3H4 Knockdown In Vitro

**Figure 2:**
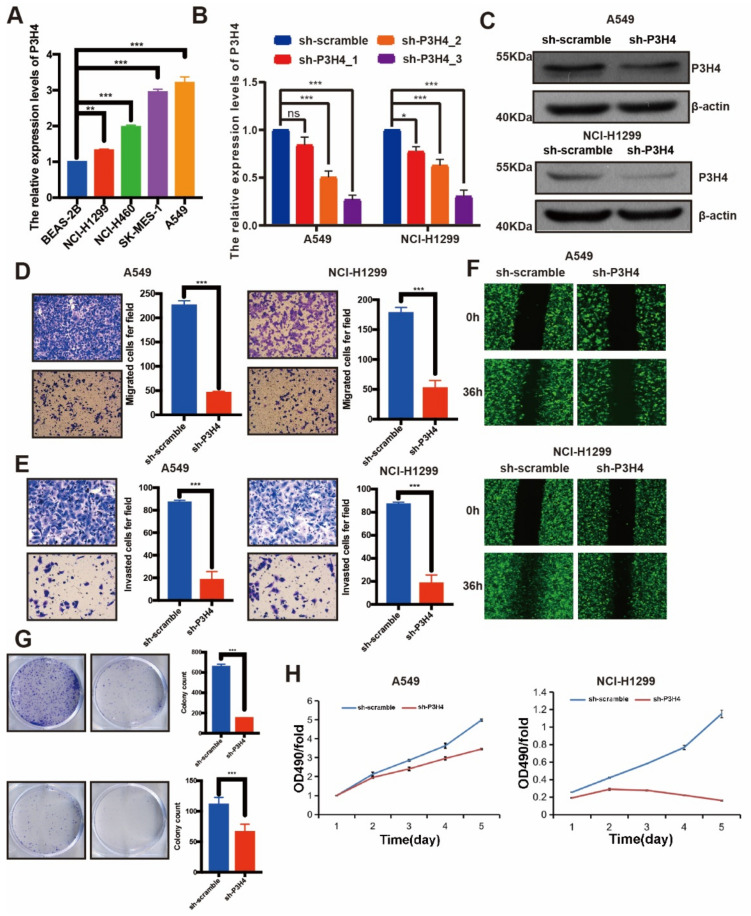
P3H4 promotes malignant progression of LUAD in vitro. (**A**) Endogenous expression of P3H4 in normal bronchial epithelial cells (BEAS-2B) and LUAD cell lines (*** *p* < 0.001, ** *p* < 0.01 vs. BEAS-2B, multiple-comparison two-ANOVA, *n* = 3). (**B**,**C**) QRT-PCR and Western blots indicate the efficiency of lentivirus in P3H4 knockdown (* *p* < 0.05, *** *p* < 0.001, vs. sh-scramble, multiple-comparison two-ANOVA, *n* = 3). (**D**–**F**) The migration and invasion of LUAD cells are suppressed by P3H4 knockdown in Transwell, Matrigel and wound healing assays (*** *p* < 0.001 vs. sh-scramble, two-tailed unpaired Student’s *t* test, *n* = 3). (**G**–**H**) The colony formation and MTT assays demonstrate decreased proliferation of LUAD cells by P3H4 (*** *p* < 0.001 vs. sh-scramble, two-tailed unpaired Student’s *t* test, *n* = 3). Original blots see Supplementary File S1.

The endogenous expression of P3H4 was identified by qRT-PCR. Higher levels of P3H4 were expressed in LUAD cell lines than in normal bronchial epithelial cells (BEAS-2B). P3H4 was highly enriched in NCI-H1299 and A549 cells (Figure 2A). The P3H4 knockdown lentivirus was constructed to suppress the expression of P3H4 in vitro. The efficiency of the lentivirus was verified in NCI-H1299 and A549 cells (Figure 2B,C). Transwell, Matrigel and wound healing assays indicated a significant decrease in the migration and invasion of A549 and NCI-H1299 cells following P3H4 knockdown (Figure 2D–F). The proliferation of A549 and NCI-H1299 cells was also suppressed by P3H4 knockdown (Figure 2G,H).

### 3.3. P3H4 Knockdown Suppressed the Malignant Progression of LUAD In Vivo

**Figure 3:**
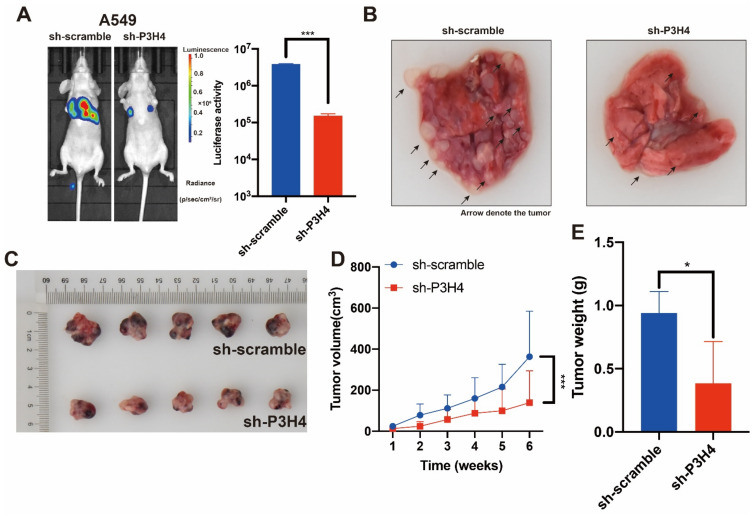
The malignant progression of LUAD is suppressed by P3H4 knockdown in vivo. (**A**) The metastasis of A549 cells was suppressed by P3H4 knockdown in vivo. (**B**) The metastatic nodes are indicated by arrows (*** *p* < 0.001 vs. sh-scramble, two-tailed unpaired Student’s *t* test, *n* = 5). (**C**–**E**) The proliferation of A549 cells is decreased by sh-P3H4 lentivirus (*** *p* < 0.001 vs. sh-scramble, multiple-comparison two-way ANOVA, *n* = 6. * *p* < 0.05 vs. sh-scramble, two-tailed unpaired Student’s *t*-test, *n* = 5).

To explore the effects of P3H4 in vivo, BALB/c nude mice were treated with P3H4 knockdown A549 cells and paired control cells via tail vein injection. The findings suggested that lung metastasis was dramatically decreased by P3H4 knockdown (Figure 3A,B). A xenograft model of BALB/c nude mice was established by implanting A549 cells with sh-circIMMP2L lentivirus and negative control (sh-scramble), and the deletion of P3H4 decreased tumor growth drastically (Figure 3C–E).

### 3.4. P3H4 Interacts with EGFR to Promote Malignant LUAD

**Figure 4:**
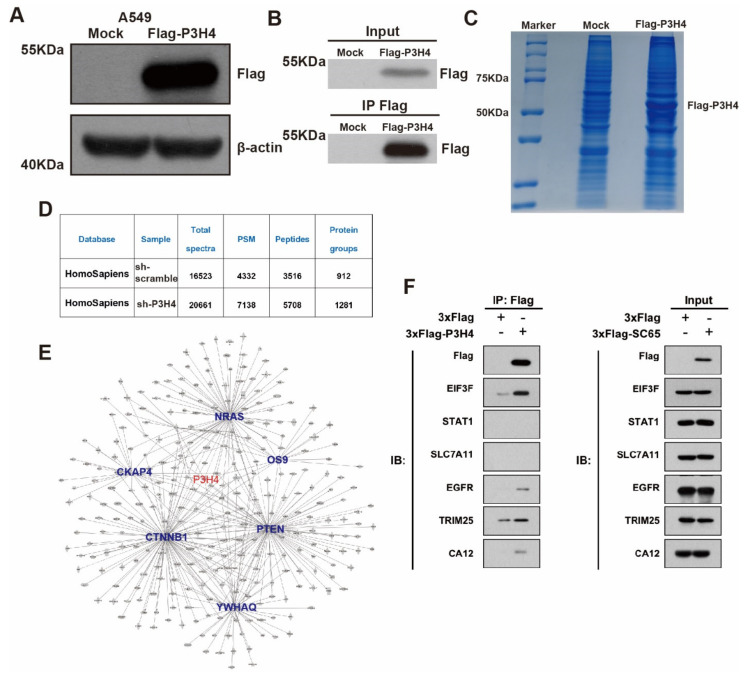
P3H4 interacts with EGFR to promote malignant LUAD. (**A**) Western blot revealed significant expression of FLAG-P3H4 lentivirus in A549 cells. (**B**) The efficiency of IP assay was identified. (**C**) Results of Coomassie Brilliant Blue staining are presented. (**D**) Summary of LC-MS/MS results. (**E**) Bioinformatics analysis of LC-MS/MS established protein interaction networks. (**F**) Six candidate interaction proteins of P3H4 were assessed via Western blot. Original blots see Supplementary File S1.

**Figure 5:**
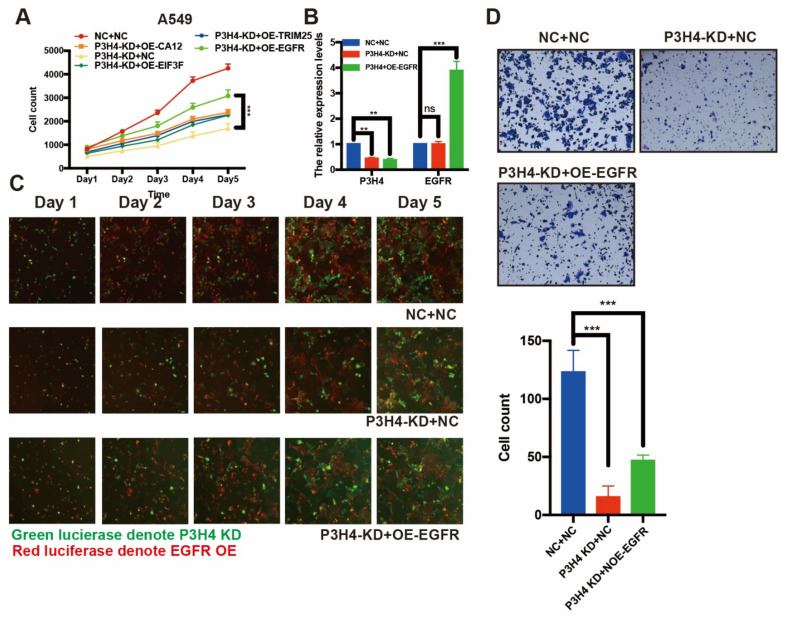
EGFR rescue the effects of P3H4 knockdown in LUAD cells. (**A**) The expression of EFGR blocked the suppression of proliferation induced by P3H4 knockdown (*** *p* < 0.001 vs. P3H4-KD + OE-EGFR, multiple-comparison two-way ANOVA, *n* = 3). (**B**) QRT-PCR revealed the efficiency of P3H4 knockdown and EGFR overexpression (*** *p* < 0.001, ** *p* < 0.01 vs. NC + NC, multiple-comparison two-way ANOVA, *n* = 3). (**C**) The proliferation of A549 cells upon P3H4 knockdown and EGFR overexpression lentivirus infection. (**D**) The representative images indicate that EGFR blocked the migration suppression following P3H4 knockdown (*** *p* < 0.001 vs. NC + NC, multiple-comparison two-way ANOVA, *n* = 3).

To explore the mechanism of P3H4 in LUAD malignant progression, we performed an immunoprecipitation (IP) assay. The FLAG-P3H4 was efficiently expressed in A549 cells (Figure 4A). The FLAG-P3H4-overexpressing A549 cells and comparable negative controls were used to perform the IP assay using the FLAG antibody (Figure 4B). Coomassie Brilliant Blue staining revealed effective capture of FLAG-P3H4 (Figure 4C). Liquid chromatography-mass spectrometry (LC-MS) was used to identify the proteins interacting with P3H4: 912 and 1281 proteins were verified in the negative control and FLAG-P3H4 groups, respectively (Figure 4D). We screened the proteins interacting only with FLAG-P3H4, while the interaction between P3H4 and other proteins was analyzed via STRING and represented in the protein–protein interaction (PPI) network (Figure 4E). Based on their association with tumorigenesis, the interaction of six proteins with P3H4 was validated. The Western blot revealed the effective interaction between EGFR and P3H4 (Figure 4F). Further, we investigated the biological function of P3H4 and six candidate proteins. We co-transfected P3H4 knockdown and candidate proteins overexpressed by a lentivirus in A549 cells. Only EGFR overexpression most significantly inhibited the proliferation induced by suppression of P3H4 knockdown (Figure 5A). P3H4 was significantly decreased and EGFR was effectively overexpressed in the assay (Figure 5B). The representative images revealed persistent proliferation of the negative control, P3H4 knockdown and P3H4 knockdown + EGFR-overexpression groups (Figure 5C). Simultaneously, the expression of EGFR also rescued the migration of A549, which was caused by P3H4 knockdown (Figure 5D).

Taken together, these results indicate that P3H4, at least partly, promoted the malignant progression of LUAD by interacting with EGFR.

P3H4 might promote the malignant progression of LUAD by regulating the metabolic pathway.

**Figure 6:**
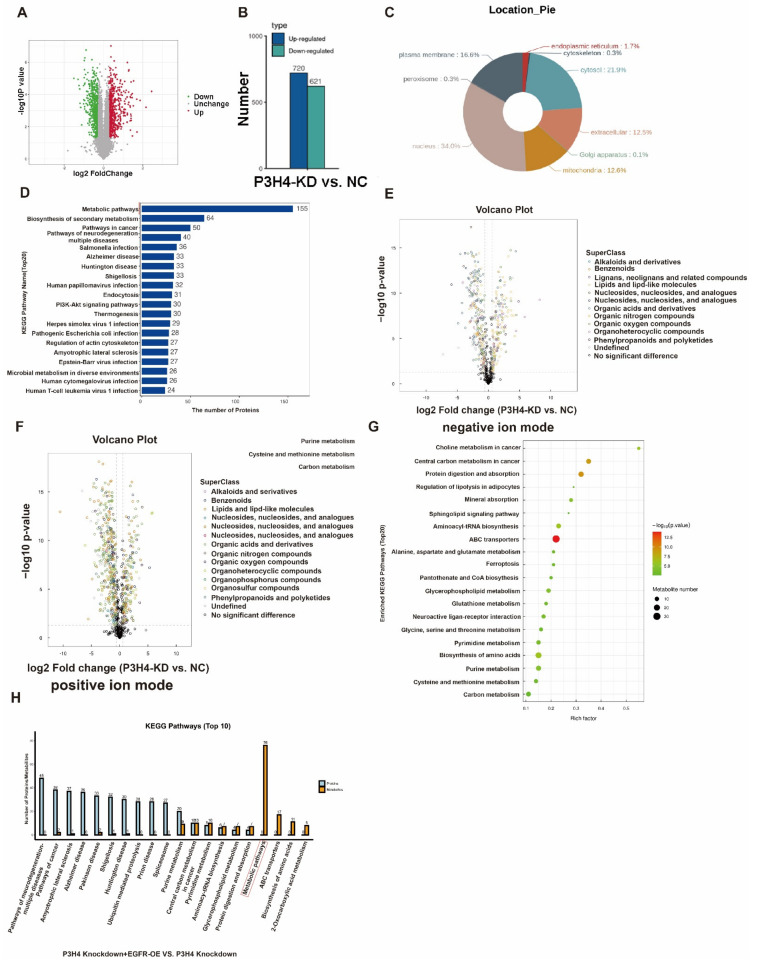
Pathway analysis of P3H4 downstream proteins. (**A**) Tandem Mass Tag (TMT) analysis of the differential expression of proteins following P3H4 knockdown. (**B**) Quantification of differential expression of proteins upon P3H4 knockdown. (**C**) The circular chart indicates the location of differentially expressed proteins. (**D**) Analysis of the KEGG pathway of P3H4-regulated proteins. (**E**) The differential expression of metabolic substances based on volcano plot using UHPLC-Q-TOF MS in negative ion mode. (**F**) The differential expression of metabolic substances based on the volcano plot using UHPLC-Q-TOF MS in positive ion mode. (**G**) Bioinformatics analysis of the KEGG pathway of differentially expressed metabolic substances following P3H4 knockdown. (**H**) Analysis of the KEGG pathway of comprehensively analyzed the proteomic and metabolomic data between P3H4 knockdown + EGFR-OE group and P3H4 knockdown group.

To explore the downstream regulation pathway, a Tandem Mass Tag (TMT) proteomics analysis of the negative control and P3H4 knockdown A549 cells was performed. The results revealed 720 upregulated and 621 downregulated genes following P3H4 knockdown (Figure 6A,B). The nuclear proteins account for the major portion of the total proteins (Figure 6C). The KEGG pathway of differential proteins regulated by P3H4 demonstrated that P3H4 might be involved primarily in the metabolism (Figure 6D). Further, we assessed the metabolic differences of A549 cells with P3H4 knockdown using untargeted metabolomics in negative and positive ion modes, suggesting the regulation of multiple metabolic substances by P3H4 (Figure 6E,F). The metabolic substances were enriched in choline metabolism in cancer and central carbon metabolism in cancer pathways (Figure 6G). We comprehensively analyzed the proteomic and metabolomic data of the P3H4 knockdown group and the group with P3H4 knockdown and EGFR overexpression. The results showed that the KEGG metabolic pathway was significantly enriched in the group with P3H4 knockdown and EGFR overexpression. This result suggests that the role of P3H4 in metabolome regulation is at least partially dependent on EGFR. Therefore, we believe that EGFR is an important P3H4-binding protein involved in metabolism (Figure 6H).

These results indicated that P3H4 promoted the malignant progression of LUAD via the metabolic pathway.

## 4. Discussion

In this study, we explored the role of P3H4 in LUAD. P3H4 was highly expressed in LUAD. Loss-and-gain functional assays revealed that P3H4 significantly facilitated the metastasis and proliferation of LUAD both in vitro and in vivo. We also investigated the possible role of P3H4 in LUAD via interaction with EGFR to regulate the metabolic pathway.

Epidermal growth factor receptor (EGFR) belongs to the ErbB family of receptor tyrosine kinases (RTKs) and exerts critical functions in epithelial cell physiology [16,17,18]. It is frequently mutated or overexpressed in different types of human cancer and is the target of multiple cancer therapies currently adopted in clinical practice [19]. Somatic activating mutations in EGFR are the most common driver mutations for which targeted therapies in NSCLC are available, and occur in 16% of patients with advanced LUAD [20]. The active mutant EGFR oncoproteins promote oncogenesis via the MAPK, PI3K–AKT and Janus kinase (JAK)–signal transducer and activator of transcription (STAT) signaling pathways [21,22].

Our studies suggest that EGFR, as an important partner of P3H4 metabolism, is at least partially involved in the P3H4 metabolic pathway. EGFR was an effective therapeutic target in non-small cell lung carcinoma. EGFR mutations and overexpression played a role in cancer cell proliferation, metastasis, chemotherapy resistance and also metabolism in NSCLC [23,24]. AKT is a classical pathway gene of EGFR and regulates cancer metabolism. EGFR activates AKT via EGFR/PI3K/AKT [24,25,26]. Thus, P3H4 represents a potential target in patients with lung cancer overexpressing EGFR mutations.

Tumor progression always occurs along with metabolic reprogramming [27]. Cancer cells, including LUAD, regulate their bioenergetic and biosynthetic pathways via metabolic adoptive regulation. The metabolic reprogramming may be mediated via tumor proliferation via upregulation or downregulation of glycolysis, nucleotides, lipids and amino acids [22,28,29]. It is well-known that proliferating cancer cells prefer uptake of glucose and secrete carbon as lactate even in the presence of oxygen [30]. Somatic mutations, such as KRAS and TP53, regulate nucleotide synthesis by reprogramming metabolism [31,32]. In our study, we demonstrated that P3H4 regulated choline metabolism and central carbon metabolism in cancer.

P3H4 is a key gene that not only regulates malignant progression of various cancers, but also participates in osteoclastogenesis and cell synthesis. The potential biological function and mechanism of P3H4 in physiological and pathological pathways require further elucidation. In our study, the interaction between P3H4 and EGFR was not clearly demonstrated, suggesting the need to investigate the pathway associated with EGFR regulation of P3H4.

## 5. Conclusions

In this study, we demonstrated that P3H4 was an important functional gene in LUAD, which was associated with advanced TNM stages and shorter survival. Upregulation of P3H4 promoted metastasis and proliferation of LUAD both in vitro and in vivo. P3H4 may combine with EGFR and regulate the metabolic substances. This study reveals a potential biomarker and therapeutic target of LUAD.

## Data Availability

The data presented in this study are available in this article and Supplementary Material.

## Supplementary Materials

The following supporting information can be downloaded at: https://www.mdpi.com/article/10.3390/cancers14133243/s1, File S1: Original blots.
