# Supplementary figures and images for: P3H4 Promotes Malignant Progression of Lung Adenocarcinoma via Interaction with EGFR
Source: Cancers (Basel). 2022 Jul 1;14(13):3243. doi: 10.3390/cancers14133243 (PMC9264976; doi:10.3390/cancers14133243)

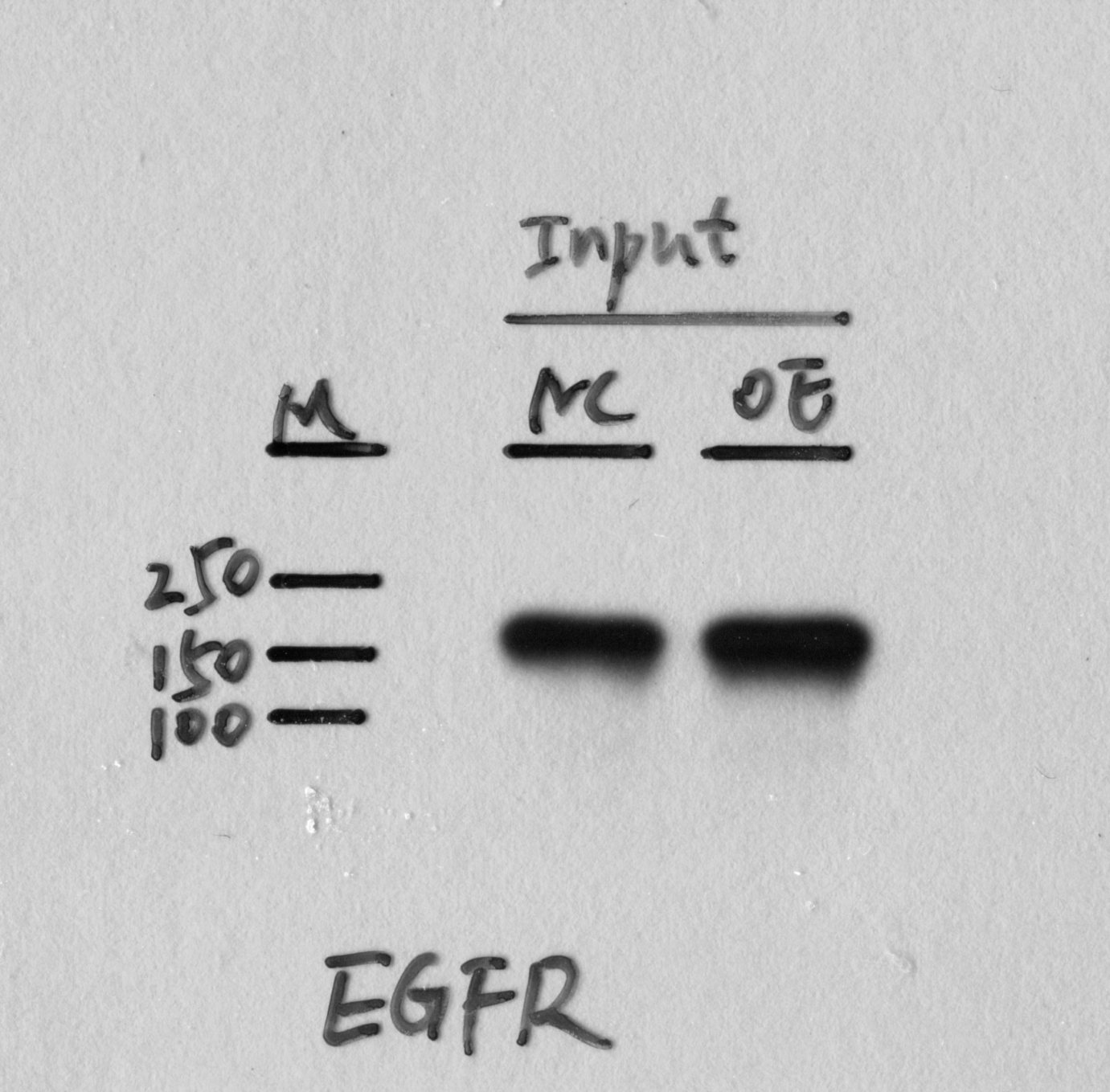

Supplement: Supplementary file 1 [file cancers-14-03243-s001.zip › Supplementary File S1/EGFR Input.jpg]

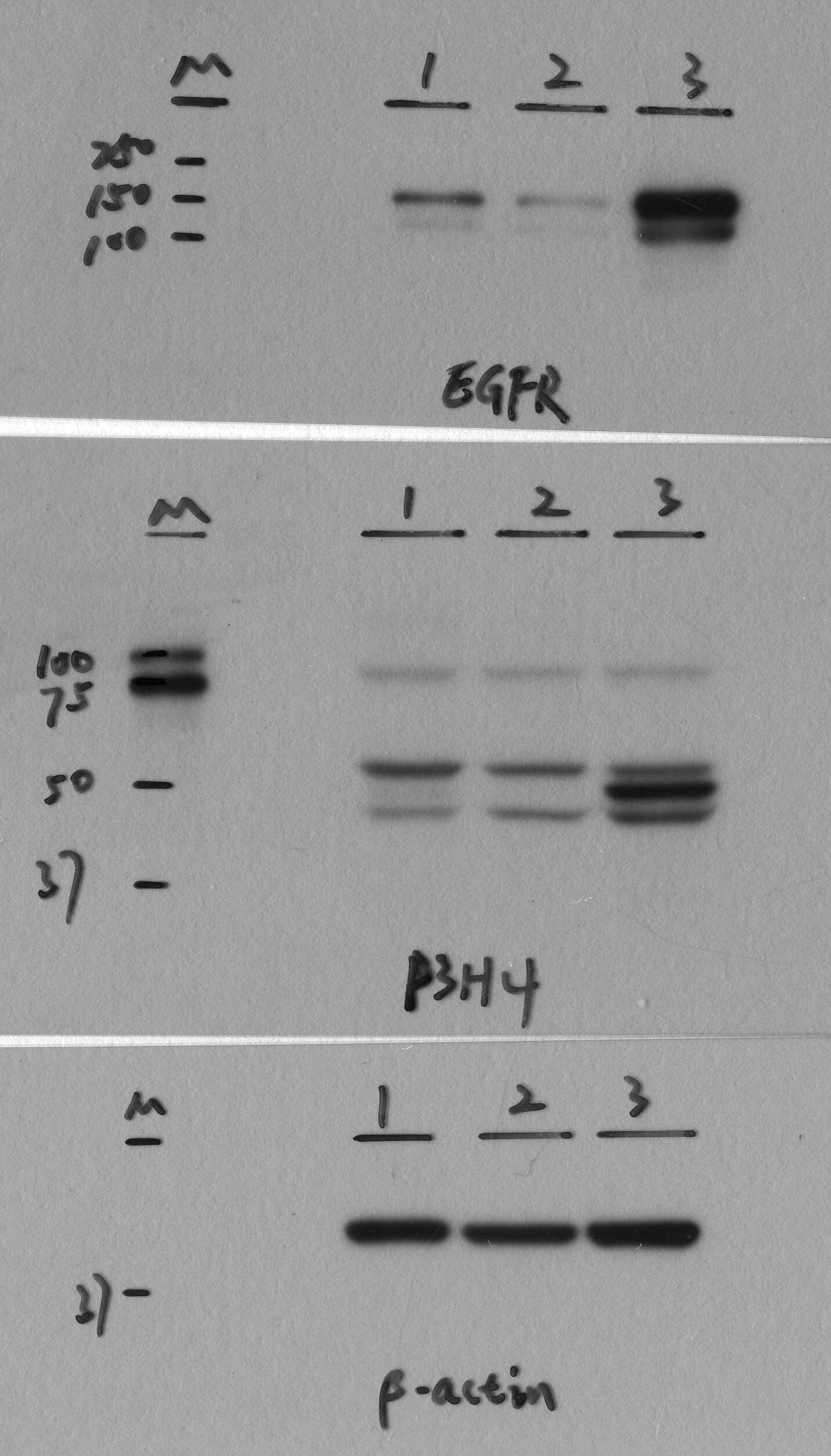

Supplement: Supplementary file 1 [file cancers-14-03243-s001.zip › Supplementary File S1/EGFR P3H4.jpg]

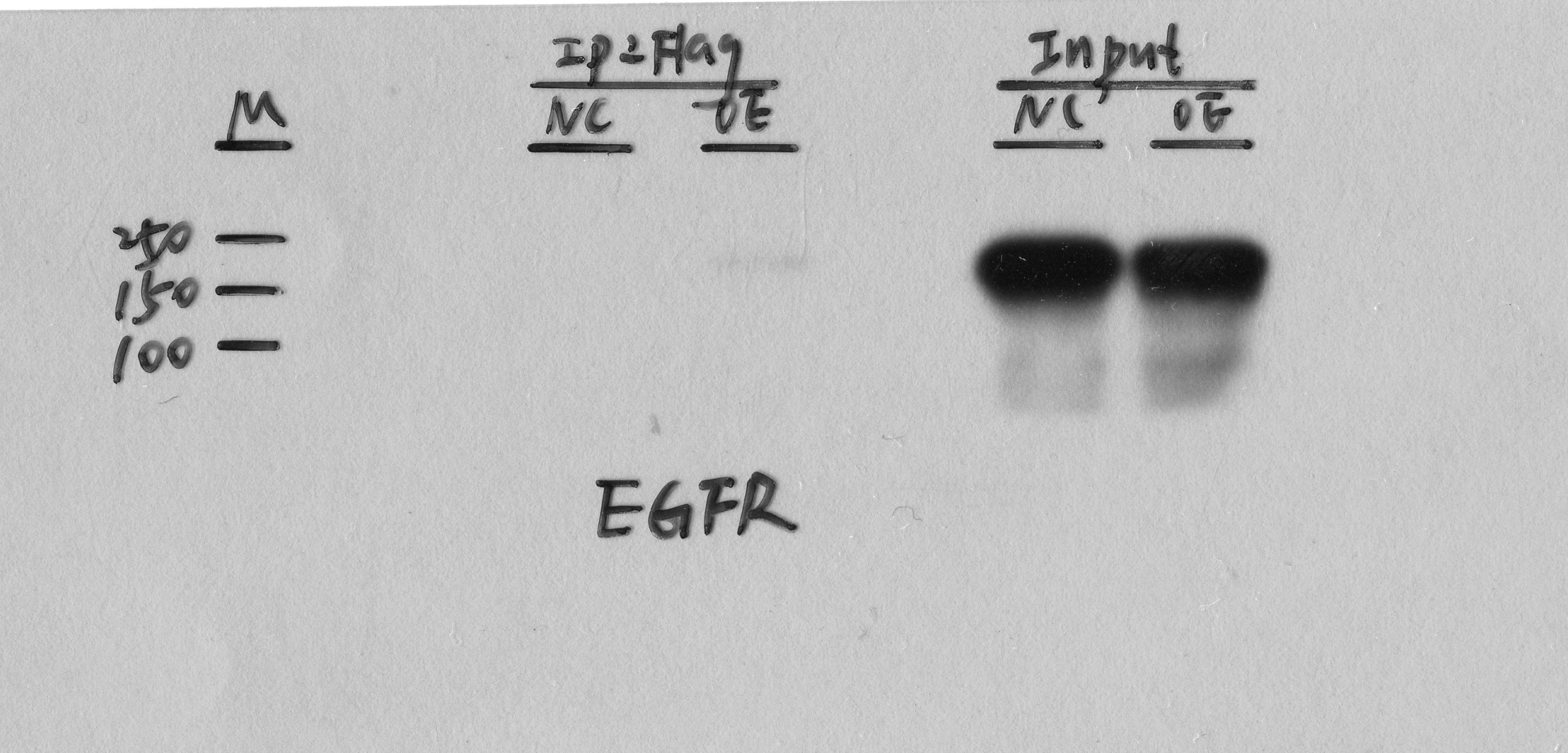

Supplement: Supplementary file 1 [file cancers-14-03243-s001.zip › Supplementary File S1/EGFR.jpg]

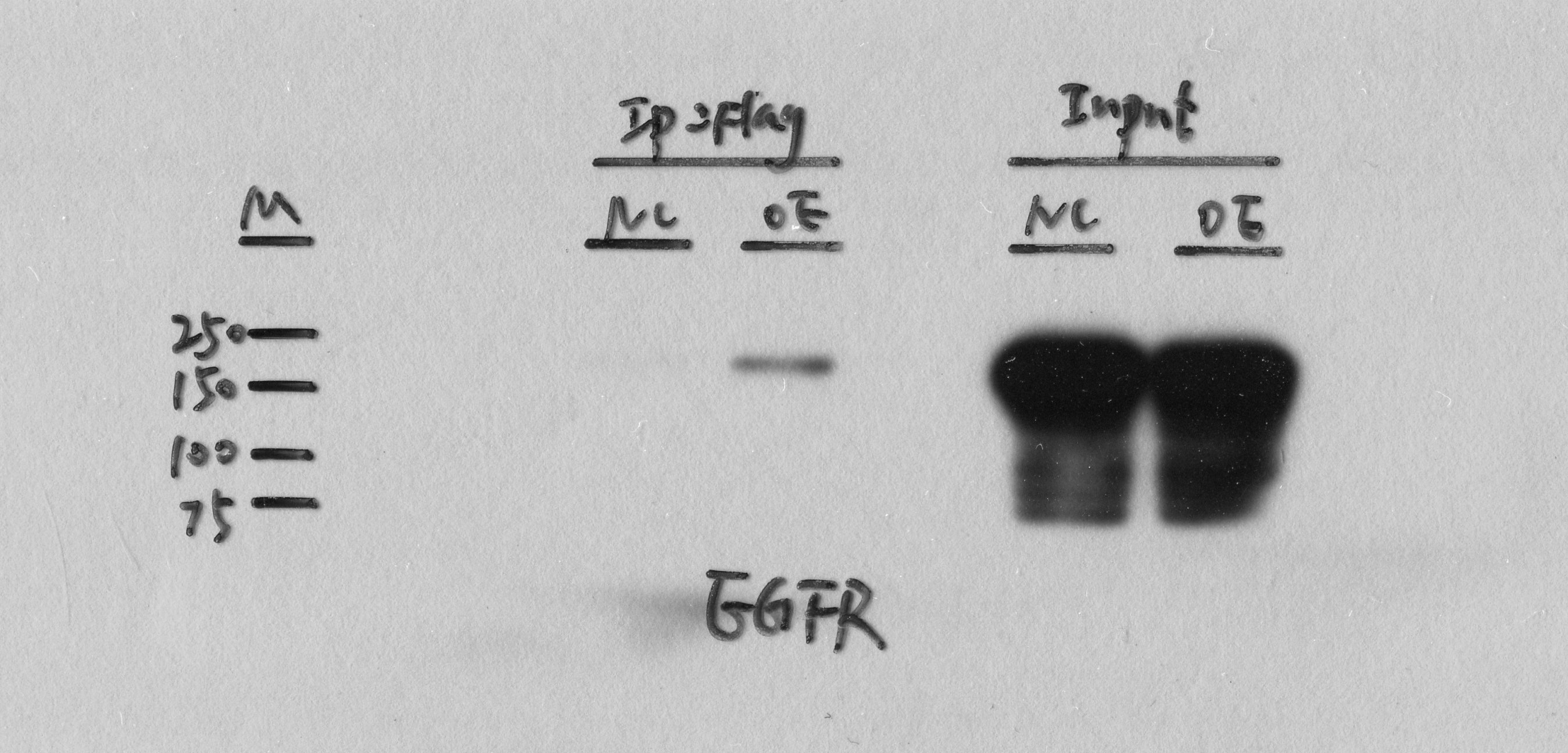

Supplement: Supplementary file 1 [file cancers-14-03243-s001.zip › Supplementary File S1/EGFRú¿╟┐ú⌐.jpg]

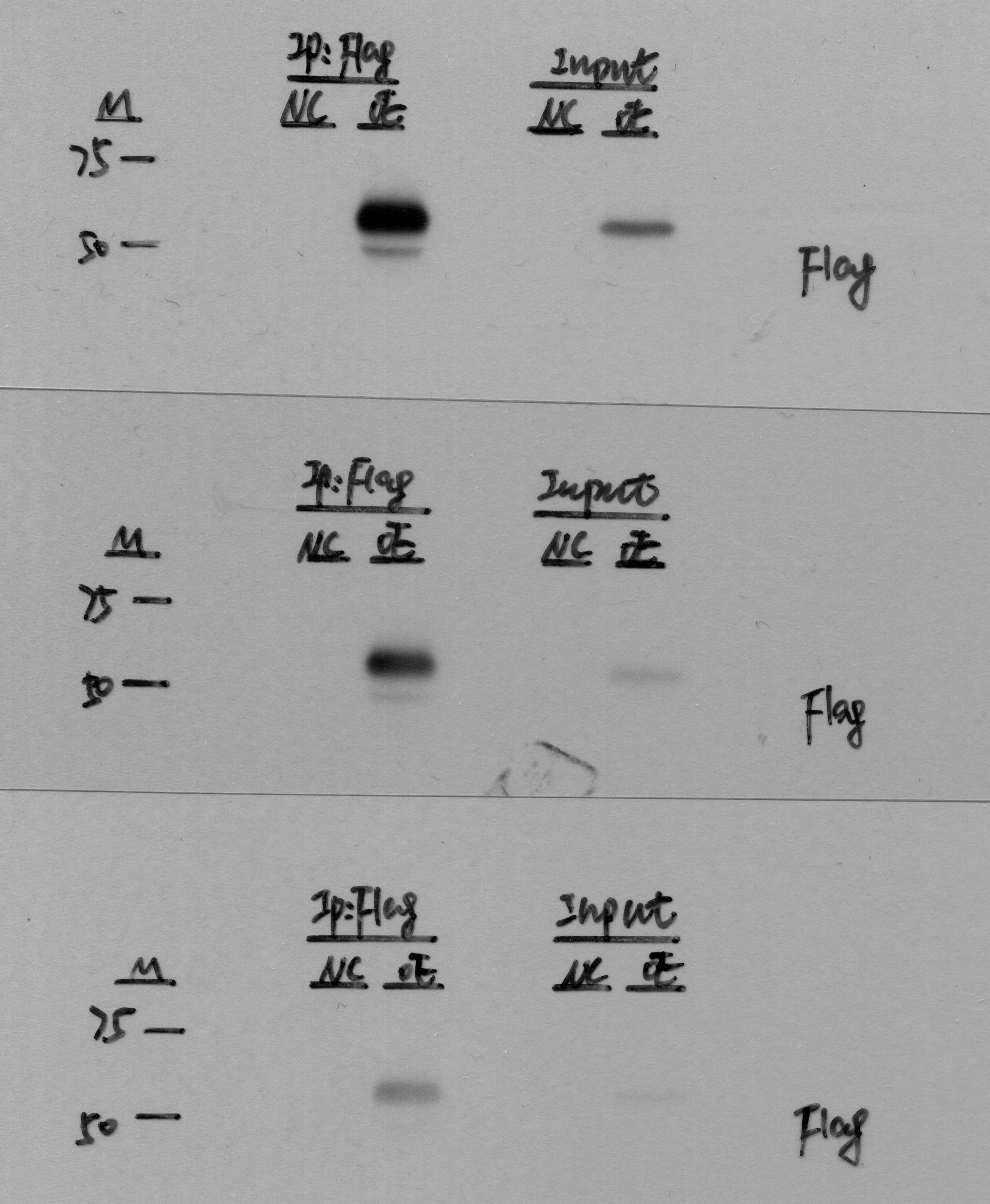

Supplement: Supplementary file 1 [file cancers-14-03243-s001.zip › Supplementary File S1/Flag.jpg]

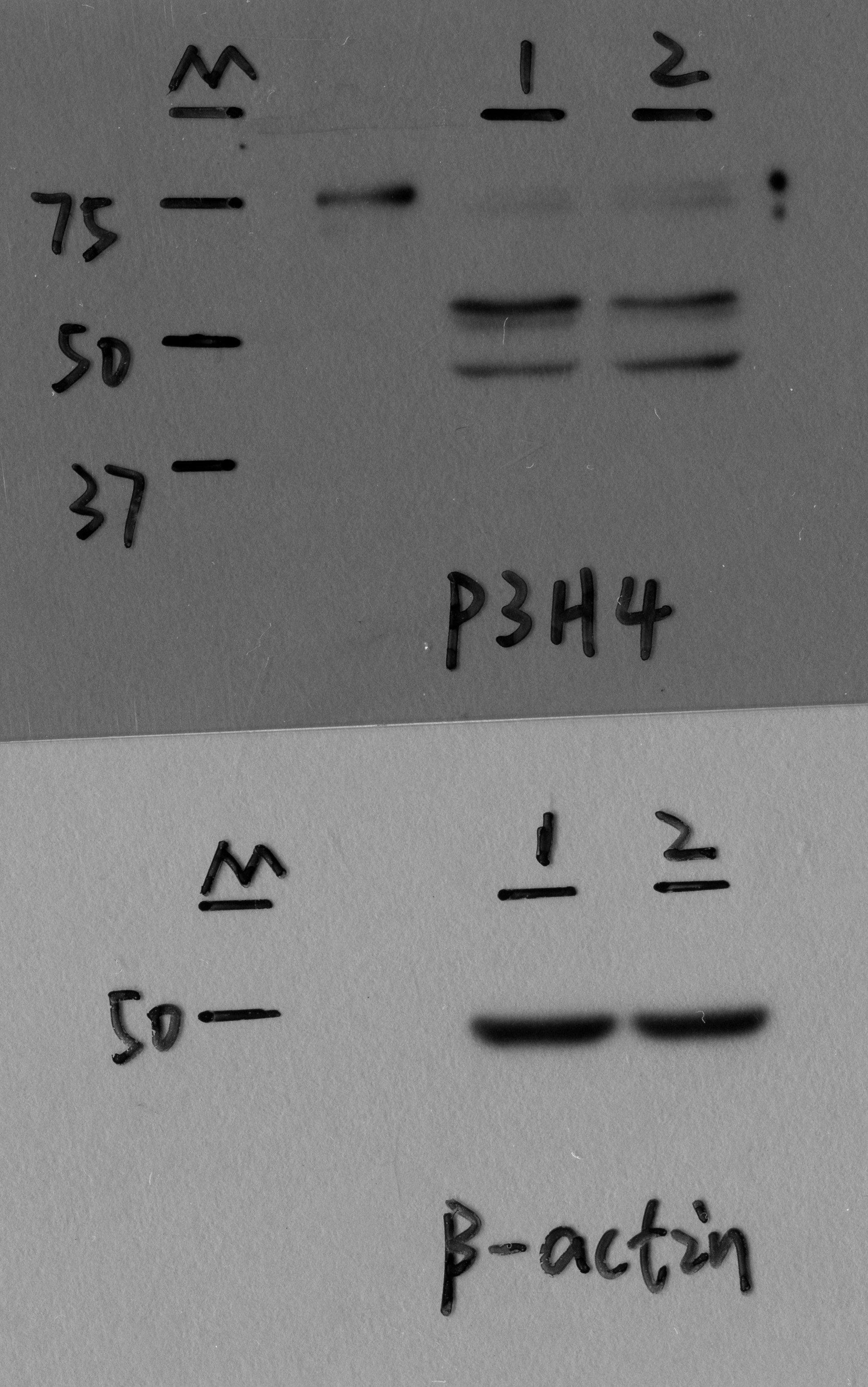

Supplement: Supplementary file 1 [file cancers-14-03243-s001.zip › Supplementary File S1/P3H4 A549.jpg]

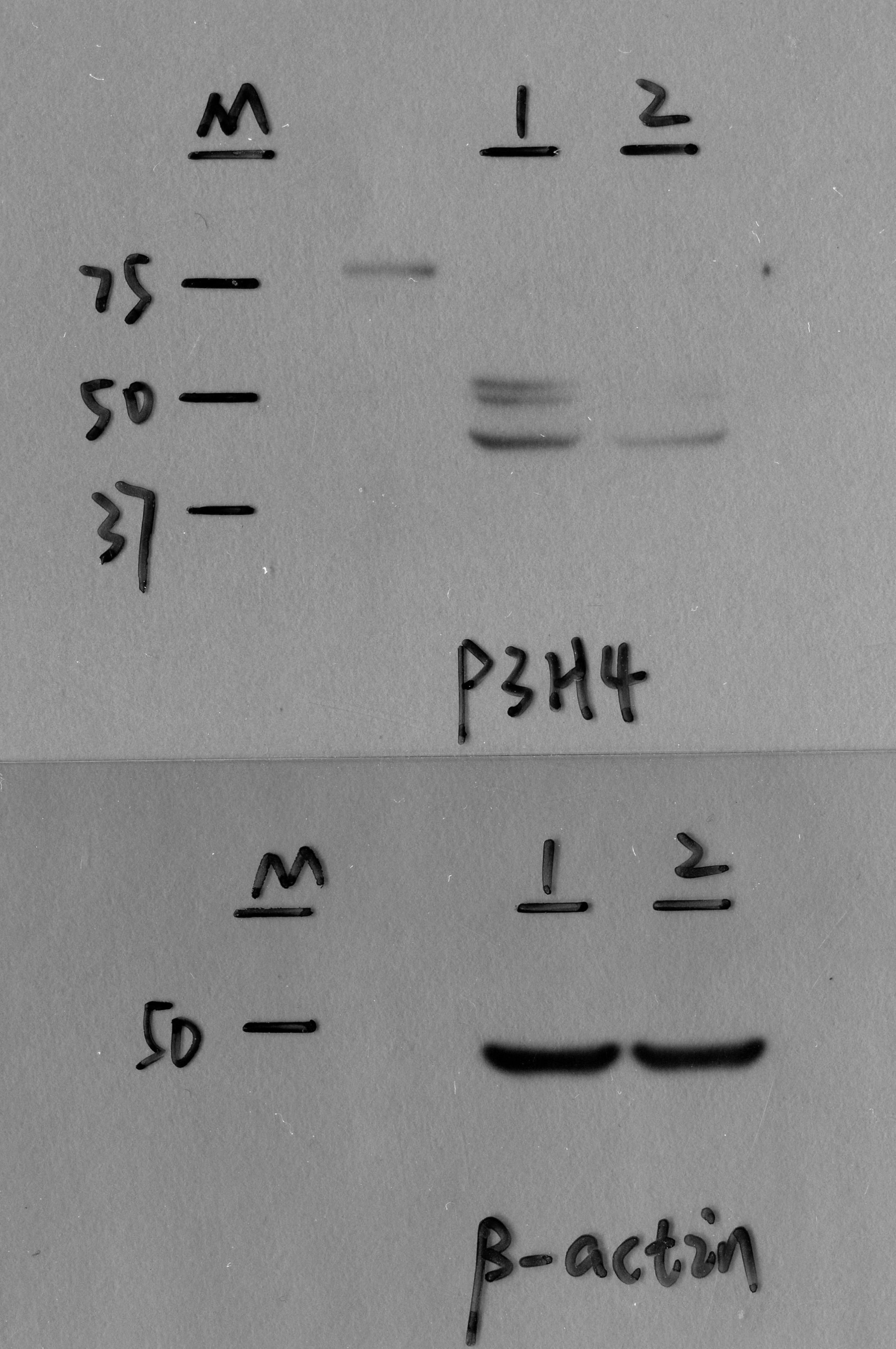

Supplement: Supplementary file 1 [file cancers-14-03243-s001.zip › Supplementary File S1/P3H4 NCI-H1299.jpg]
